# Impact of delay extubation on the reintubation rate in patients after cervical spine surgery: a retrospective cohort study

**DOI:** 10.1186/s13018-023-04008-9

**Published:** 2023-08-02

**Authors:** Xin Jing, Zhengfang Zhu, Hairong Fan, Junjie Wang, Qing Fu, Rongrong Kong, Yanling Long, Sheng Wang, Qixing Wang

**Affiliations:** Department of Critical Care Medicine, Shanghai Tenth People’s Hospital, Tongji University School of Medicine, Shanghai, 200072 China

**Keywords:** Cervical spine surgery, Risk factors, Extubation, Reintubation

## Abstract

**Background:**

The incidence of cervical airway obstruction after cervical spine surgery (CSS) ranges from 1.2 to 14%, and some require reintubation. If not addressed promptly, the consequences can be fatal. This study investigated delayed extubation's effect on patients' reintubation rate after cervical spine surgery.

**Methods:**

We performed a retrospective case–control analysis of cervical spine surgery from our ICU from January 2021 to October 2022. Demographic and preoperative characteristics, intraoperative data, and postoperative clinical outcomes were collected for all 94 patients. Univariable analysis and multivariable logistic regression were used to analyze postoperative unsuccessful extubation risk factors following cervical spine surgery.

**Results:**

The patients in the early extubation (*n* = 73) and delayed extubation (*n* = 21) groups had similar demographic characteristics. No significant differences were found in the reintubation rate (0 vs. 6.8%, *p* = 0.584). However, the delayed extubation group had significantly more patients with 4 and more cervical fusion segments (42.9 vs. 15.1%, *p* = 0.013),more patients with an operative time greater than 4 h (33.3 vs. 6.8%, *p* = 0.004)and all patients involved C2-4 (78 vs. 100%, *p* = 0.019).Also, patients in the delayed extubation group had a longer duration of ICU stay (152.9 ± 197.1 h vs. 27.2 ± 45.4 h, *p* < 0.001) and longer duration of hospital stay (15.2 ± 6.9 days vs. 11.6 ± 4.1 days, *p* = 0.003). Univariate and multivariate analysis identified the presences of cervical spondylotic myelopathy (CSM) (OR 0.02, 95% CI 0–0.39, *p* = 0.009) and respiratory diseases (OR: 23.2, 95% CI 2.35–229.51, *p* = 0.007) as unfavorable prognostic factor for reintubation.

**Conclusions:**

Our analysis of patients with cervical spondylosis who received CSS indicated that delayed extubation was associated with the presence of respiratory diseases and CSM, longer operative time, more cervical fusion segments, and longer duration of ICU and hospital stays.

## Introduction

Cervical spondylosis (CS) is a chronic degenerative cervical spine disorder characterized by spinal cord compression and neurological deficits. It mainly includes cervical radiculopathy, cervical myelopathy, or combinations of the two. CS has increased significantly in recent decades due to lifestyle changes [1]. Cervical discectomy and fusion effectively treat cervical spine disorders [2].

Various complications may develop after cervical spine surgery (CSS), including new-onset of neurological dysfunctions, cerebrospinal fluid (CSF) leaks [3], dysphagia, hoarseness, surgical site infection, hematoma [4], axial neck pain [5], chronic neck pain [6], and others. Airway obstruction is one of the most adverse consequences after cervical operation in the first 24 h. In practice, it is recommended to extubate a patient after cervical spine surgery with greater caution and prudence [7]. On the other hand, delayed extubation may increase various mechanical ventilation-associated complications, and the patient is likely to develop ventilator-associated pneumonia, resulting in a poor prognosis. The incidence of postoperative airway obstruction following anterior CSS can be 1.2–14% [8, 9]. It can occur during the immediate postoperative period or 24 to 72 h following surgery. If the airway obstruction is not discovered early and treated appropriately, it can cause reintubation and serious unfavorable events such as pharyngeal fistulas [10], hypoxic-ischemic encephalopathy [11], cardiac arrest, and death. For patients undergoing CSS, deciding whether to extubate immediately following surgery or after a careful examination upon return to the ICU with a tube is crucial.

Based on the above reasons and clinical utility value, we retrospectively analyzed the postoperative patients admitted to the ICU after CSS. We investigated the influence of delayed extubation on the reintubation rate to improve the success rate of postoperative extubation.

## Methods

### Study design and setting

This study was approved by the Research Ethics Committee of Shanghai's tenth people's hospital (Approval number: 22k313). The data used in this study were fully anonymized before analysis. Patient consent was waived for all participants enrolled in this study because of the retrospective study design. This study followed the Strengthening the Reporting of Observational Studies in Epidemiology (STROBE) guidelines.

This single-center, retrospective, observational, and comparative cohort study was performed using data from the electronic medical records of all patients diagnosed with CS and admitted to Shanghai's tenth people's hospital between January 1, 2021, and October 31, 2022. The anesthesia clinician and the surgeon decided to admit a patient to the ICU or the ward. All patients passed a weaning assessment and spontaneous breathing trial before extubation. The attending physicians and trained respiratory therapists in our ICU made the final extubation decision.

### Definition of variables

All patients were divided into early extubation and delayed extubation groups. The early extubation group was defined as an extubated patient when transferred to ICU. Delayed extubation in this study was defined as a patient who had not been extubated when transferred to ICU.

### Data collection

We collected the following information from the medical records of both groups of patients: demographics [age, gender, weight, height, and body mass index (BMI)]; perioperative data; intraoperative data (the operative type, operative approach, operated segment, operative time, total blood loss, blood pressure, and others); and outcomes including reintubation rate, ICU and hospital length of stay.

### Outcomes

The key outcome was the rate of reintubation. Secondary outcomes included length of stay in the ICU and hospital.

### Statistical analysis

All participants who met the inclusion and exclusion criteria were included in the final analysis. Data are displayed as mean ± standard deviation for parametric data, median and interquartile range (IQR) for nonparametric data, and numbers and percentages for categorical data. Differences between continuous variables were assessed using a Student's *t* test for parametric data and a Mann–Whitney *U*‐test for nonparametric data. The Chi‐square test and Fisher exact test were used for categorical variables. Univariate and multivariate analyses using the Cox proportional hazard regression model were conducted to identify independent risk factors for the reintubation rate. A *p* value < 0.05 was set as statistical significance. All analyses were performed using R Statistical Software (http://www.R-project.org, The R Foundation) and the Free Statistics analysis platform (Beijing, China).

## Results

### Subject baseline demographic and clinical data

**Fig. 1:**
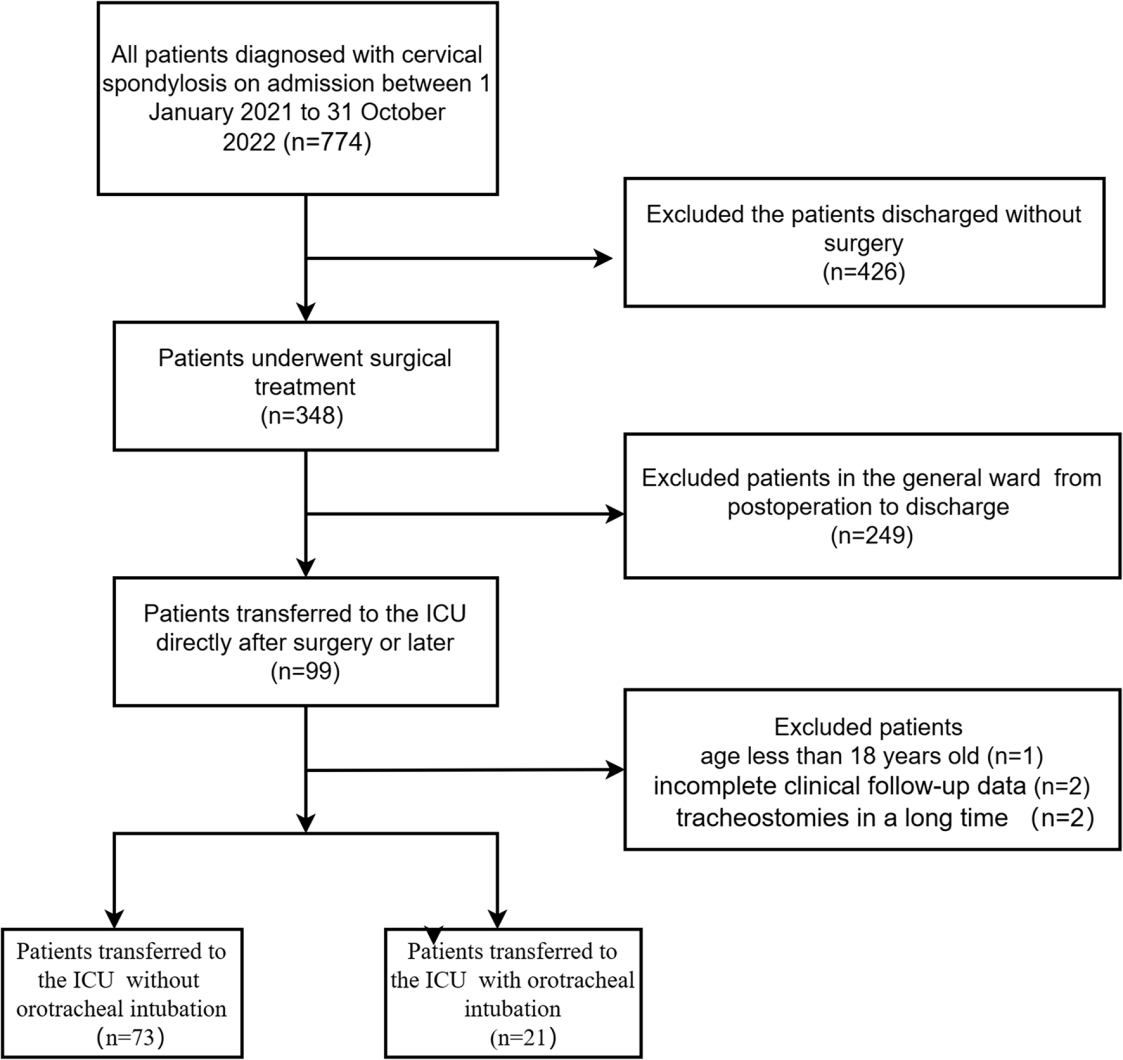
The admission process of experimental population

From January 2021 to October 2022, a total of 774 patients were diagnosed with cervical spondylosis on admission, 348 of them underwent surgical treatment; however, 249 of them were excluded from the present study due to reasons listed in Fig. 1, leaving 94 patients for the final analysis (Fig. 1). Of these, 73 patients were extubated immediately following surgery and transferred to the ICU, while 21 patients were not extubated immediately after surgery and transferred to the ICU. At baseline, the two groups were well-matched (Table 1). There were no significant differences in gender, age, BMI, and underlying disease. The mean age of both groups was 62.5 ± 11.3 years, and there were more male patients than female patients. Nearly half of the included patients had a history of hypertension (40.4%). Almost one-third of all the patients in the early extubation group had a history of diabetes mellitus (30.1%). But there were significant differences in the preoperative diagnosis (*P* < 0.01). There were 34 cases (36.2%) of cervical spondylotic radiculopathy(CSR), 50 cases (53.2%) of cervical spondylotic myelopathy(CSM), and 10 cases (10.6%) of other types such as trauma and tumor.

**Table 1:** Baseline demographic and clinical data of patients included

	Total (*n* = 94)	Early extubation (*n* = 73)	Delayed extubation (*n* = 21)	*p* value
Age (years, range)	62.5 ± 11.3	62.0 ± 11.5	64.0 ± 10.7	0.483
Gender (male, %)	58 (61.7)	45 (61.6)	13 (61.9)	0.983
BMI (kg/m^2^)	24.0 ± 2.8	24.3 ± 2.7	23.1 ± 3.2	0.101
Underlying disease				
Hypertension (%)	38 (40.4)	32 (43.8)	6 (28.6)	0.209
Diabetes (%)	24 (25.5)	22 (30.1)	2 (9.5)	0.056
Cerebrovascular disease (%)	6 (6.4)	3 (4.1)	3 (14.3)	0.123
Respiratory system disease (%)	17 (18.1)	14 (19.2)	3 (14.3)	0.755
Coronary heart disease (%)	4 (4.3)	2 (2.7)	2 (9.5)	0.215
Smoking (%)	15 (16.0)	9 (12.3)	6 (28.6)	0.093
Diagnosis				< 0.001*
CSR	34 (36.2)	26 (35.6)	8 (38.1%)	
CSM	50 (53.2)	44 (60.3)	6 (28.6%)	
Other (trauma, tumor)	10 (10.6)	3 (4.1)	7 (3.33%)	

*BMI* body mass index, *CSR* cervical spondylotic radiculopathy, *CSM* cervical spondylotic myelopathy

*statistically significant

### Characteristics of intraoperative and outcomes

The intraoperative conditions of the patients in both groups are shown in Table 2. ACDF was performed in half of the patients (53.2%). Due to the large number of elderly patients and trauma patients, posterior approach surgery accounted for a large proportion (30.8%). But there was no difference in two groups. More patients underwent the anterior cervical operation in both groups. There was a statistically significant difference in cervical fusion segment levels. More patients in the delayed extubation group had more than four cervical fusion segments (15.1 vs. 42.9%, *p* = 0.013). When comparing both groups, a significant difference was found for the surgical procedure time (*p* = 0.004). In the delayed extubation group, all the patients’ involved segment contain C2-4 (78 vs. 100%, *p* = 0.019). There was no difference in intraoperative blood loss for both groups (*p* = 0.149). Regarding intraoperative hypotension, the number of patients did not differ between the groups.

**Table 2:** Intraoperative data between the groups included in the final analysis

	Total (*n* = 94)	Early extubation (*n* = 73)	Delayed extubation (*n* = 21)	*P* value
Operative type				0.179
ACCF	12 (12.8)	11 (16.1)	1 (4.8)	
ACDF	50 (53.2)	41 (56.2)	9 (42.9)	
ACCF + ACDF	3 (3.2)	2 (2.7)	1 (4.8)	
Other (posterior, fixation)	29 (30.8)	19 (26)	10 (47.6)	
Operative approaches (anterior, %)	68 (72.3)	55 (75.3)	13 (61.9)	0.225
Fusion segment levels (4 segments, %)	20 (21.3)	11 (15.1)	9 (42.9)	0.013*
Hours of operative time (> 4, %)	12 (12.8)	5 (6.8)	7 (33.3)	0.004*
Estimated blood loss (> 300 ml, %)	35 (37.2)	30 (41.1)	5 (23.8)	0.149
C2-4 (yes, %)	78 (83.0)	57 (78.0)	21 (100)	0.019*
Intraoperative hypotension	22 (23.4)	18 (24.7)	4 (19)	0.772
Plantation (yes, %)	69 (73.4)	56 (76.7)	13 (61.9)	0.261

*ACCF* anterior cervical corpectomy and fusion, *ACDF* anterior cervical discectomy and fusion

*statistically significant

### Patient outcomes

**Fig. 2:**
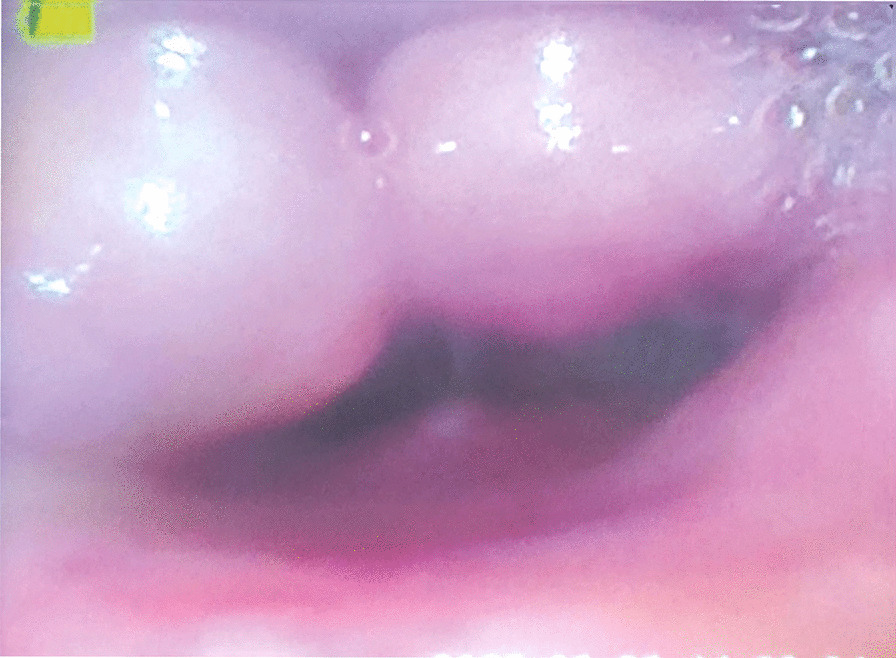
Laryngeal edema by laryngoscopy

The results showed that the primary outcome and the reintubation rate did not differ significantly between the groups (p  = 0.584) (Table 3). However, there were no reintubated patients in the delayed extubation group. In the 5 cases of reintubation, the reintubation occurred within 48 hours after opration, with a mean of 15.05h and a median of 10h. While in the delayed extubation group, the mean extubation time was 41.53 h after surgery, and the median was 22h. All the reintubated patients presented acute upper airway obstruction and were diagnosed with laryngeal edema using laryngoscopy (Fig. 2). Compared to that in the early extubation group, the ICU stay of patients was significantly higher in the delayed extubation group (27.2 ± 45.4 h vs. 152.9 ± 197.1 h, p＜0.001).However,the two groups had no significant difference in hospitalized days.  The cuff leak test (CLT) was routinely used to assess laryngeal edema prior to extubation. The sensitivity and specificity of cuff leak test used a cutoff of 110 mL, a value that is frequently used in clinical practice [12].In our study, the volume of cuff-leak in the delayed extubation group patients was approximately 45% of inspiratory tidal volume. Some patients who passed the cuff-leak test still had slight pharyngeal edema as observed by bronchoscopy or laryngoscopy.

**Table 3:** Outcomes between the groups included in the final analysis

	Total (*n* = 94)	Early extubation (*n* = 73)	Delayed extubation (*n* = 21)	*P* value
Length of ICU stay, h	55.3 ± 112.8	27.2 ± 45.4	152.9 ± 197.1	< 0.001*
Length of hospital stay, days	12.4 ± 5.0	11.6 ± 4.1	15.2 ± 6.9	0.003*
Reintubation rate (%)	5 (5.3)	5 (6.8)	0 (0)	0.584
Time to reintubation (h, mean ± SD)		15.05 ± 14.82		
Time to extubation (h, mean ± SD)			41.53 ± 36.31	

*statistically significant

When analyzing the link between the significant association factors and reintubation rate with univariable logistic regression, we found that only preoperative respiratory system disease was associated with reintubation (Table 4). The multivariable analysis found that preoperative respiratory system disease was independently associated with reintubation. The patients with CSM will increase the risk of delayed extubation (OR 0.02, 95% CI 0–0.39). The respiratory system disease will also statistically increase the risk of delayed extubation (OR 23.2, 95% CI 2.35–229.51).

**Table 4:** Univariable and multivariable analysis of factors affecting reintubation rate

Variables	Univariable analysis		Multivariable analysis	
	OR (95% CI)	*p* value	OR (95% CI)	*p* value
Age (cont. var.)	1.08 (0.97~1.2)	0.15		
Gender: female versus male	0 (0~Inf)	0.995		
BMI (cont. var.)	1.15 (0.83~1.59)	0.411		
Delayed intubation: yes versus no	0 (0~Inf)	0.994		
Diagnoses				
CSR versus others	0.25 (0.03~2.06)	0.197	0.08 (0.01–0.95)	0.045
CSM versus others	0.08 (0.01~1.01)	0.051	0.02 (0–0.39)	0.009*
Underlying disease				
Hypertension: yes versus no	0.35 (0.04~3.27)	0.358		
Diabetes: yes versus no	0.72 (0.08~6.75)	0.772		
Cerebrovascular disease: yes versus no	4.2 (0.39~44.92)	0.235		
Respiratory system disease: yes versus no	23.38 (2.42~226.11)	0.006*	23.2 (2.35~229.51)	0.007*
Coronary heart disease: yes versus no	0 (0~Inf)	0.994		
Smoking: yes versus no	0 (0~Inf)	0.993		
Operation route				
ACDF versus ACCF	26,754,554.64 (0~Inf)	0.995		
ACCF + ACDF versus ACCF	1 (0~Inf)	0.999		
Others versus ACCF	12,574,640.68 (0~Inf)	0.995		
Intraoperative period				
Operative approaches: anterior versus posterior	0.64 (0.07~6.01	0.696		
Fusion segment levels: less versus 4 segments	2.63 (0.41~16.93)	0.309		
Hypotension: yes versus no	0.81 (0.09~7.64)	0.854		
Hours of operative time > 4: yes versus no	5.27 (0.78~35.44)	0.088		
Estimated blood loss > 300 ml: yes versus no	1.13 (0.18~7.12)	0.895		
Plantation: yes versus no	1.39 (0.15~13.12)	0.772		
C2-4:yes versus no	21,531,918.89 (0~Inf)	0.995		

*statistically significant

### The clinical detail of 5 patients with reintubation

This article summarized the clinical detail of 5 cases of reintubation in the early extubation group (Table 5). In the table, we can find these patients had more similar points, such as the sex, the age and the more segments**.**

**Table 5:** The clinical detail of 5 patients with reintubation

No.	Sex	Age, year	BMI	Fluid balance	Seg	Op. indication	Medical comorbidity	Op. type	Op. time min	LP	EBL, mL	Time to reintubation hr	Time to extubation, hr	Reason	Losicu (h)	Los (day)
1	M	68	27.68	2350	C3-5	Trauma	Diabate /Respdis	ACCF	121	No	100	10	72.5	No cough & massive sputum	117	10
2	M	70	22.15	1450	C3-7	CSR	Hypertension /Braindis/ Respdis	ACDF Fixation	241	No	150	41.25	120	Hematoma	177	18
3	M	68	25.61	4050	C2-7	CSM	No	Posterior approach for atlantoaxial fusion	353	No	1500	5	15.5	Upper Airway obstruction	16.5	25
4	M	73	23.89	450	C3-6	CSR	Respdis	ACDF	233	No	400	8	78.3	Hematoma	81.3	23
5	M	69	25.95	1500	C4-6	CSM	Respdis	ACDF	170	Yes	200	11	105	OSAS & airway edam	354	24

*LP* lowpress (SBP ≤ 90 mmHg),  *EBL* estimated blood loss

## Discussion

With medical technology's development, the cervical fusion level required in CSS has become increasingly complex. The more intraoperative strain on the trachea and its surrounding tissues leads to increased uncertainty about surgical damage to the airway and edema in the pharynx. Prolonged intubation and unplanned reintubation are associated with a greater rate of postoperative complications and mortality [13]. Because of the small probability of postoperative complications in cervical spondylosis, retrospective cohort studies are predominant. In the available retrospective studies, numerous factors causing prolonged mechanical ventilation and reintubation of tracheal intubation are highly variable and still controversial, thus need to be studied.

Our study found that although there was no statistically significant difference in reintubation rates with delayed and early extubation, none of the patients in the delayed extubation group experienced reintubation. In patients with delayed extubation, we strictly follow a combination of bronchoscopy and the balloon leak test to assess the patency of the patient's upper airway to determine whether to extubate the artificial airway. By standardizing the extubation procedure in this way, the possibility of reintubation is avoided. It has been reported that the mortality rate increased proportionately to the interval between extubation and reintubation [14, 15].

A case of tracheotomy excluded from the inclusion process of this study was a patient who developed a hematoma within 12 h after being transferred to a general ward after immediate postoperative extubation and failed to intervene in time to cause cardiac and respiratory arrest after resuscitation to restore voluntary heartbeat and respiration. However, brain function remained in an irreversible vegetative state, causing a great tragedy to the patient's family and hospital. Therefore, we may be inclined to believe that a comprehensive review is necessary before extubation following CSS to avoid reintubation. This parallels the research perspectives of Mishra et al. [4].

In addition, our study found that the duration of surgery longer than 4 h is a high-risk factor for delayed extubation, consistent with previous study results [7, 16]. The tip of the tracheal tube is close to the inner airway wall, and mechanical stimulation induces significant congestion and edema of the airway mucosa, particularly in the prone position during posterior approach surgery. We reviewed the bronchoscopic records of the patients in the delayed extubation group who underwent posterior cervical spine surgery and found that 3 cases of severe pharyngeal edema and 2 cases of minor edema were clearly documented. Figure 2 shows a patient undergoing posterior cervical fusion with severe arytenoid cartilage edema. After prolonged surgery, the damage to the anterior cervical soft tissues increases and is more likely to cause postoperative anterior soft tissue swelling, which is the second most common cause of postoperative acute airway obstruction besides hematoma. Think about the operator, as the duration of surgery increases, there is a certain degree of decrease in the operator's concentration and refinement of movements, resulting in damage to the pericervical tissues or damage to the superior and recurrent laryngeal nerves, both of which can cause vocal fold dyskinesia and increased edema of the posterior pharyngeal wall tissues.

The number of CSS segments is another important factor influencing delayed extubation in patients. Marquez et al. [17] found that the reintubation rate after an elective anterior cervical fusion was 0.5%, increasing to 1.6% after more than 3 level fusions. This study found that the delayed extubation group had more patients with more than four cervical fusion segments [18]. Hence, the likely reason is that when the procedure is performed with anterior decompression and bone graft fusion (ACDF), the operator will perform a blunt separation along the cervical vessels between the cervical vessels and the tracheoesophageal to access the anterior cervical fascia. Blunt separation tends to stress and pull on the trachea, resulting in ischemic edema of the peritracheal tissue. The greater the number of segments operated on, the more tissue is mechanically stretched, resulting in a large area of edema in the superior and inferior vocal tissues. Respiratory illness was identified as an additional risk contributing to prolonged extubation and reinsertion. Patients with chronic obstructive pulmonary disease (COPD) have a poor base of lung function and a high airway reactivity that predisposes them to airway spasm; the mechanical stimulation of surgery induces an inflammatory response in the airways, leaving the patient's upper and lower airways at the peak of the inflammatory response for 72 h after surgery, followed by an increase in the patient's airway secretions, which further decreases the ventilation and diffusion function of the lungs [19]. In our study, there was two patients had the history of OSAS. It had been shown that a 49-year-old female patient exhibited a marked exacerbation of OSAS subsequent to undergoing ACDF. This phenomenon could potentially be attributed to the impairment of the pharyngeal plexus and the constriction of the posterior plate placement within the cervical region, resulting in the collapse and stenosis of the airway [20]. At the same time, we think it is related to postoperative analgesia and sedation. Hence, postoperative extubation in patients undergoing cervical spine surgery with the preoperative underlying respiratory disease requires proper evaluation and preparation, and delayed extubation may be more beneficial to the patient. The diagnose of the CSM also is the most important independent risk factors. In the guidelines for CSM, age-related degeneration is the primary cause of CSM. Most CSM patients were older. Those with > 3 levels involved, cervical stenosis, posterior compression, or congenital stenosis would likely benefit from a posterior approach, which also has a greater amount of blood loss during decompression. Perioperative management is more important. For perioperative patients with CSM, the two most common complications were cardiopulmonary problems (3.3%) and dysphagia (3.0%) [21]. Delayed extubation can ensure adequate rest after surgery, reduce the burden on heart and lung, and avoid adverse reactions caused by throat edema.

The median reintubation time was 10 h in the 5 patients in the early extubation. The median extubation time was 22 h in the delayed extubation group. Therefore, we recommend that patients with high-risk factors (such as CSM, respiratory complications, surgical level > 4, etc.) could stay in the ICU for intensive care with mechanical ventilation for 18–20 h. Then, through the spontaneous breathing trial (SBT), cuff-leak-test, tracheoscopy, the intensive care unit physician and respiratory therapist make the decision to extubation.

In the current study, we found that delayed extubation can significantly prolong the postoperative length of ICU and hospital stays, and our findings were consistent with previous studies [22, 23]. Although hospitals have standard procedures for weaning and extubation, there is a possibility that a delay may b}e due to the final decision on extubation being made by intensivists [24]. As a result, patients with delayed extubation spend more time in the ICU, with a corresponding increase in the chance of ICU-related complications and, ultimately, a longer hospital stay.

There were several limitations of this study. First, it was a single-center retrospective analysis. Although the baseline characteristics of the two groups were similar, there may be other uncontrolled confounding variables in the study that we have not considered. Therefore, we need a multicenter randomized controlled trial (RCT) to verify our results. Second, there are no gold-standard criteria for extubation after CCS, particularly for patients with respiratory disease. Hence, the timing of extubation is partly based on the anesthesiologist or attending intensivist's subjective judgment. Finally, there was no consensus on the need for routine laryngoscopy to check for laryngeal edema before extubation. More relevant studies will be required in the future to draw more reliable conclusions.

## Conclusion

In summary, the current study demonstrates that patients with an operating duration of more than 4 h, more than four operative segments, and respiratory diseases should be prioritized by the surgeon, who should carefully decide whether to extubate immediately after surgery. The extubation of such patients involves a careful evaluation of the upper airway. We also need to assess postoperative respiratory muscle strength, the amount of secretions, and the ability to contour the airway in patients with trauma-induced spinal cord damage. Further research is required to develop optimal extubation protocols for patients undergoing cervical spine surgery so that extubation is more precise and reintubation is avoided.
